# Brain Responses to Food Choices and Decisions Depend on Individual Hedonic Profiles and Eating Habits in Healthy Young Women

**DOI:** 10.3389/fnut.2022.920170

**Published:** 2022-06-24

**Authors:** Nicolas Coquery, Yentl Gautier, Yann Serrand, Paul Meurice, Elise Bannier, Ronan Thibault, Aymery Constant, Romain Moirand, David Val-Laillet

**Affiliations:** ^1^INRAE, INSERM, Univ Rennes, CHU Rennes, Nutrition Metabolisms and Cancer Institute, NuMeCan, Rennes, France; ^2^Inria, CRNS, Inserm, IRISA UMR 6074, Empenn U1228, Univ Rennes, Rennes, France; ^3^CHU Rennes, Department of Radiology, Rennes, France; ^4^Unité de Nutrition, CHU Rennes, Rennes, France; ^5^EHESP, School of Public Health, Rennes, France; ^6^Unité d’Addictologie, CHU Rennes, Rennes, France

**Keywords:** eating behavior, decision-making, brain, healthy subjects, fMRI, rsfMRI

## Abstract

The way different food consumption habits in healthy normal-weight individuals can shape their emotional and cognitive relationship with food and further disease susceptibility has been poorly investigated. Documenting the individual consumption of Western-type foods (i.e., high-calorie, sweet, fatty, and/or salty) in relation to psychological traits and brain responses to food-related situations can shed light on the early neurocognitive susceptibility to further diseases and disorders. We aimed to explore the relationship between eating habits, psychological components of eating, and brain responses as measured by blood oxygen level-dependent functional magnetic resonance imaging (fMRI) during a cognitive food choice task and using functional connectivity (FC) during resting-state fMRI (rsfMRI) in a population of 50 healthy normal-weight young women. A Food Consumption Frequency Questionnaire (FCFQ) was used to classify them on the basis of their eating habits and preferences by principal component analysis (PCA). Based on the PCA, we defined two eating habit profiles, namely, prudent-type consumers (PTc, *N* = 25) and Western-type consumers (WTc, *N* = 25), i.e., low and high consumers of western diet (WD) foods, respectively. The first two PCA dimensions, PCA1 and PCA2, were associated with different psychological components of eating and brain responses in regions involved in reward and motivation (striatum), hedonic evaluation (orbitofrontal cortex, OFC), decision conflict (anterior cingulate cortex, ACC), and cognitive control of eating (prefrontal cortex). PCA1 was inversely correlated with the FC between the right nucleus accumbens and the left lateral OFC, while PCA2 was inversely correlated with the FC between the right insula and the ACC. Our results suggest that, among a healthy population, distinct eating profiles can be detected, with specific correlates in the psychological components of eating behavior, which are also related to a modulation in the reward and motivation system during food choices. We could detect different patterns in brain functioning at rest, with reduced connectivity between the reward system and the frontal brain region in Western-type food consumers, which might be considered as an initial change toward ongoing modified cortico-striatal control.

## Introduction

Food choices and decisions shape our eating habits and are determinants of individual trajectories in terms of wellbeing, health, and disease susceptibility. Individual eating habits can develop and evolve according to psychological profiles, life events, and environments. In the context of a nutritional pathology or established eating disorder (ED), daily food choices are frequently explored to identify deleterious habits that should be modified as part of medical care. However, how different food consumption habits in healthy individuals with no ED or any other pathological condition can shape the emotional and cognitive relationships with food and further disease susceptibility is much less investigated. Even people who present a “normal” eating behavior (i.e., with no pathological features) can present small or important variations in terms of preferred and selected food items. These variations, in the context of repeated more or less healthy daily food choices, might favor the onset of different neurocognitive individual profiles possibly associated with different risks for further disorders and diseases.

Palatable and high-energy (HE) food accessibility is currently considered to be one of the major causes of the development of EDs and obesity. Chronic exposure to highly palatable food (i.e., fatty, sweet, and processed foods) and/or exposure during critical developmental stages is correlated with hyperphagia or craving, which could ultimately lead to disturbances of the energy homeostasis and nutritional balance (1). Several studies have compared the effect of fatty/sweet food abuse (i.e., chronic consumption associated or not with obesity) and the effect of drugs with the brain reward circuit, the consumption behavior, and mental health and general health (2, 3). Brain responses and behavior related to hedonia (notably the dopaminergic system) are different between subjects with obesity, who are overweight, and with normal-weight (4–7). However, it is still not known whether the chronic consumption of palatable foods (independent of weight gain or initial health problems) can induce a shift in the hedonic and cognitive processes that regulate food intake and choices.

The *model of dynamic vulnerability of obesity* (8–10) tends to integrate the principal theories associating reward and obesity. This model suggests that chronic palatable food consumption provokes a dynamic phenomenon that leads to a shift in hedonic and cognitive neurobehavioral processes regulating food intake. According to the *reward surfeit theory* (9), people with initial neural vulnerability present a reward hypersensitivity toward palatable food perception, which would lead to increased food consumption (habitual consumption) and weight gain. Through behavioral conditioning mechanisms, habitual consumption of palatable food induces hypersensitivity of brain regions related to attention and hedonic evaluation toward stimuli usually predicting food reward—*incentive sensitization theory* (11–13), which promotes hyperphagia. Consequently, excessive palatable food consumption can induce a negative regulation of the reward dopaminergic system in the striatum, as well as in the inhibitory centers involved in the food stimulation response, such as the prefrontal cortex. According to the *reward deficit theory* (14), these modifications will contribute to the acceleration of overconsumption and to the emergence of EDs or obesity.

Currently, the concept of an individual trajectory toward health and diseases has gained strong interests in the context of personalized medicine. Early identification of subjects at risk of developing a disease allows for early prevention strategies (15). In normal eaters without any pathology, classical measurements [body mass index (BMI), glycemia, etc.] and declarative information (e.g., ED and psychological questionnaires) are usually not sufficient to describe an individual phenotype to predict future risks related to specific eating habits. Concomitant analysis of eating behavior and cognitive factors, with dedicated neuroimaging-based brain function analyses, would represent a potent approach to decipher the decision-making mechanisms underlying food choices and life habits, as well as their health consequences (16).

In a preliminary study (17), we validated a food frequency questionnaire-based approach aiming at identifying subtle variations in terms of eating behavior in a normal young eater population, with a focus on the consumption of Western-type food items, i.e., fatty, sweet, salty, and/or processed foods. We demonstrated in a small number of subjects that this strategy is a valid approach to identify different consumption profiles correlated with food hedonic orientations (i.e., high vs. low consumers of Western-type foods). Subgroups within a population can be compared in terms of hedonic orientations toward Western-type food items, assessed through a liking evaluation cognitive task, and also in relation to different psychological traits and eating behavior components, assessed with dedicated questionnaires, such as the Three-Factor Eating Questionnaire Revised, 18-item (TFEQ-R18) (18). Using a validated visual cognitive food choice task in the context of functional magnetic resonance imaging (fMRI) in healthy volunteers with different eating profiles would represent a potent strategy to identify specific neurocognitive features associated with eating habits and preferences or with particular behavioral traits such as cognitive restraint, uncontrolled eating, and emotional eating. For example, a previous study identified the modifications in brain connectivity involved in the cognitive control of eating in the context of obesity (19), but data are lacking to document these modifications in healthy normal-weight subjects with contrasted eating habits.

In this study, we aimed to investigate whether we could characterize an ongoing trajectory toward maladapted eating behavior as determined by chronic hedonic food consumption in relation to the components of eating behavior and brain functions in a healthy normal-weight young population, with no ED or any other pathology. We specifically focused on the cortico-striatal pathway as a sentinel of cognitive control efficacy related to food choices. We deliberately selected a population of young women who are known to be more compliant with experimental protocols and for whom the probability of risky eating habits and EDs is higher than that in men. Considering our recruitment potential for this study (*N* = 50) and the inter-gender variability in terms of brain functioning, selecting only women was also a means to reduce interindividual variability for brain imaging analyses.

## Materials and Methods

### Ethics Statement

This study was approved by an Independent National Research Ethics Committee (Comité de Protection des Personnes “Ile de France II,” project N° 2017-03-03; N° ID-RCB/EUDRACT 2017-A00133-50; National Clinical Trial number: NCT03076489) and was performed in accordance with the French and European General Data Protection Regulation 2016/679.

### Participants

#### Participants

**FIGURE 1:**
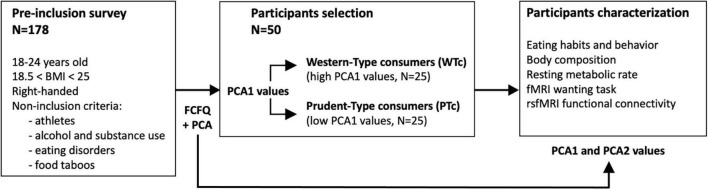
Overall study design. With the principal component analysis (PCA) performed on the Food Frequency Questionnaire (FCFQ) data obtained from 178 volunteers, 50 participants were selected by the values of the first PCA axis (PCA1) that discriminated Western-type from prudent-type consumers (WTc, with high PCA1 values vs. PTc, with low PCA1 values). The first two axes of the PCA (PCA1 and PCA2) were used to further characterize the 50 selected participants.

From a pre-inclusion survey consisting of 178 participants, 50 healthy women living in France for at least 1 year were selected based on their eating habits as previously described (17) (see below for further details). The participants met the following inclusion criteria: between 18 and 24 years old, normal BMI between 18.5 and 25, and right-handed. All the participants were native French speakers and provided their free and informed consent to participate in this study. The overall study design is presented in Figure 1.

#### Recruitment

Participants were excluded if they were athletes, reported substance use (tobacco, alcohol, drug, etc.) or EDs (e.g., bulimia, anorexia, or binge ED), food taboos based on ideological (vegetarianism and veganism), religious, or health (allergy and intolerances) motives. Pregnant women, people without French health insurance, and/or insufficient knowledge of the French language were also not included. These selection criteria were chosen to recruit a relatively homogenous group of healthy volunteers without any addiction or ED, who could understand the instructions clearly and evaluate the food pictures without any extreme beliefs or taboos related to food consumption.

Participants were selected using the following self-administered paper questionnaires. The Ricci & Gagnon questionnaire was used to assess physical activity levels, and participants were excluded in case of high physical activity level represented by a score >32 (20). The Alcohol Use Disorders Identification Test (AUDIT) questionnaire was used to assess drinking patterns, and we excluded excessive drinkers defined as score >7 for women and >8 for men (21). The Car, Relax, Alone, Forget, Friends, Trouble (CRAFFT) questionnaire was used to assess substance-related risks and problems in adolescents, and we excluded participants if the score was >2, which indicated “yes” (22). The Sick, Control, One stone, Fat, Food (SCOFF) questionnaire was used to assess the possible presence of an ED, and participants were excluded if the score was >1, which indicated “yes” (23). Daily smokers were excluded based on answers to questions related to smoking and e-cigarette consumption because of the nicotine impact on eating behavior and sensory abilities, while occasional smokers were included (consumption frequency <1/day).

#### Eating Habit-Based Group Definition

Group definition was adopted from our previous study (17). Briefly, we used the Food Consumption Frequency Questionnaire (FCFQ) (24) in which we chose to keep only the palatable food items (*N* = 56, no picture, text only) that are usually associated with risky eating habits and for which the consumption frequency must remain low according to the French PNNS (Programme National Nutrition Santé – National Nutrition and Health Program). These items were grouped in seven categories (cold meat–eggs, meat, processed food, cheese and dairy products, appetizers, sweet, ice cream, and fat) as previously described (17). This questionnaire assessed food-eating frequencies during the last 12 months asking for each food item “*In the past 12 months, how often did you consume*…*?*” The frequency responses were defined and scored as follows: never (scored 0), fewer than one time a month (scored 0.5), one to three times a month (scored 2), one time a week (scored 4), two to five times a week (scored 14), one time a day (scored 30), and several times per day (scored 60), corresponding to a monthly consumption frequency.

**FIGURE 2:**
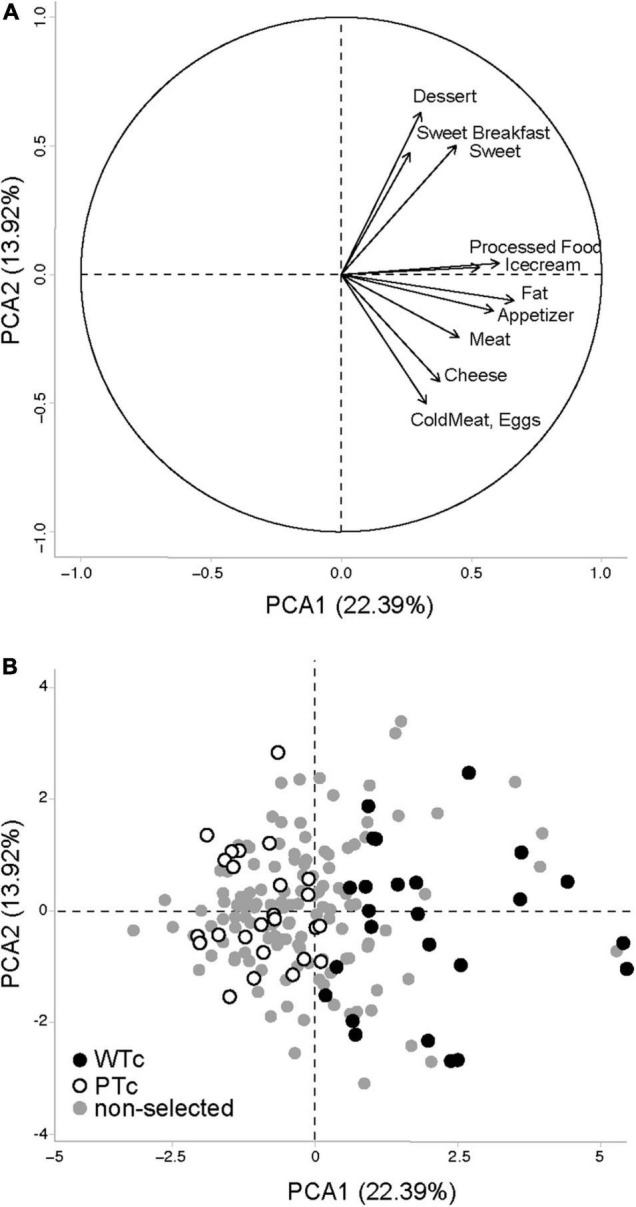
Principal component analysis (PCA) performed on the Food Frequency Questionnaire data obtained from 178 volunteers, with the **(A)** projection vectors of the different food items included in the questionnaire focused on Western-type foods, and **(B)** individual values projected on the first two axes of the PCA. WTc, Western-type consumers (*N* = 25) with the highest values for PCA1 among the 50 volunteers selected for the brain imaging study. PTc, prudent-type consumers (*N* = 25) with the lowest values for PCA1 among the 50 volunteers selected for the brain imaging study. The first two axes of the PCA bear, respectively, 22.39 and 13.92% of the population variability.

The responses were analyzed by principal component analysis (PCA; Figure 2). The two first dimensions (PCA1 and PCA2) brought complementary descriptions of the selected population. We separated the participant population into two equal groups using the projected value on dimension 1 (PCA1): Western-type consumer (WTc) group, *n* = 25, with the highest consumption of transformed fat and sweet items, and prudent-type consumer (PTc) group, *n* = 25, with the lowest consumption of transformed fat and sweet items. For further fMRI and rsfMRI analyses, we modeled the data with the projected value on both PCA dimensions in order to highlight the discrete distribution of the effect of eating habits.

#### Eating Behavior Characterization

The Three-Factor Eating Questionnaire-Revised 18-item (TFEQ-R18) measures cognitive and behavioral components of eating (18). It includes three subscales: (1) cognitive restraint (conscious restriction of food intake in order to control body weight or to promote weight loss) comprised six items (e.g., “I consciously hold back at meals in order not to gain weight”), (2) uncontrolled eating (tendency to eat more than usual due to a loss of control over intake accompanied by subjective feelings of hunger) comprised nine items (e.g., “When I see a real delicacy, I often get so hungry that I have to eat right away”), and (3) emotional eating (inability to resist emotional cues) comprised three items (e.g., “When I feel blue, I often overeat”). Internal consistency reliability coefficients (Cronbach’s α) for each of the three scales were above the 0.70 standard and below the 0.90 limit recommended for individual assessment. The responses were scored on a four-point scale, and anchors may vary across items (e.g., definitely true to definitely false, or never to at least one time a week). The raw scale scores are transformed to a 0–100 scale. Higher scores on the respective scales are indicative of greater cognitive restraint, uncontrolled eating, or emotional eating (18). The Emotional Overeating Questionnaire (EOQ) is a six-item self-report questionnaire that assesses overeating frequency in response to six emotions, namely, anxiety, sadness, loneliness, tiredness, anger, and happiness (25), previously used in a French study (26). Each item begins with “Have you eaten an unusually large amount of food given the circumstances in response to feelings of (…).” Each of the six emotions is presented in all capital letters, followed by three more synonyms in parentheses and in lower case. The response set for the six items is a 7-point scale reflecting the frequency of days in which the behavior occurred in the past 28 days (i.e., 0 = never, 1 = 1–5 days, 2 = 6–12 days, 3 = 13–15 days, 4 = 16–22 days, 5 = 23–27 days, and 6 = every day).

### Body Composition

Body composition was assessed by bioimpedance analysis using a mono frequency (50 kHz) bioimpedance analyzer (Bodystat 1500 MDD, Bodystat Ltd., Man Island, United Kingdom). The measurement procedure was complied with the standard procedure and the manufacturer’s instructions. The measurement was taken in participants’ underwear, when lying down for at least 5 min. They should not touch the cables, the impedance meter, a third party, or a metal object. Totally, four single-use electrodes were placed on the patient’s right hemibody or left hemibody: two electrodes were placed on the upper limb—one on the back of the hand and the other on the back of the wrist, and the other two electrodes were placed on the lower limb—one on the dorsal side of the foot and the other on the anterior side of the ankle. Dedicated cables were connected from the device to the appropriate electrodes.

### Resting Energy Expenditure

The indirect calorimeter used was the Quark RMR (Cosmed, Italy). The canopy was used to measure resting energy expenditure (REE) in spontaneously breathing subjects. The procedure recommended by Oshima et al. (27) was followed. The subject was placed under a clear canopy with a plastic drape to avoid air leakage. Calorimeters feature a constant flow generator to create an outward flow through the canopy. The exhaled breath by the subject is diluted by the constant flow Q (L/min) and collected by the calorimeter for gas analysis, which enables calculations of oxygen and carbon dioxide consumptions. These values are used to calculate the EE using Weir’s equation.

### Functional Magnetic Resonance Imaging Wanting Task

#### Liking (i.e., Hedonic) Evaluation of the Food Pictures

Participants completed a hedonic evaluation (17) answering the question “*Indicate how much you like this starter/main course/dessert using the numeric scale from 1 to 10 at the bottom of the screen*,” ranging from 1: “*I really don’t like it*” to 10: “*I really like it*,” for each of the 160 food pictures from our database. The responses were used to determine dyads of pictures for the fMRI wanting task.

#### Wanting Task Procedure

The general fMRI procedure was the same as described by van der Laan et al. (28), which we have already implemented as previously described (17). The grades from the liking evaluation (see above) were used to determine individual pairing of food pictures for the fMRI wanting task in order to create two conditions: the similar liking (SL) condition that should represent a conflicting food choice situation and the different liking (DL) condition that should represent a non-conflicting food choice situation.

In the SL, as in the DL conditions, there was always a factor-2 caloric gap (within the 1 to 10 numeric scale) between associated pictures determining a HE vs. a low-energy (LE) food choice (as shown in Figure 1). In the DL condition, the food item with the highest liking score (i.e., the preferred food) was always the HE food item. Each two-choice task in the fMRI was consequently individually designed on the basis of individual hedonic ratings.

### Brain Activation Analysis

#### Preparation of the Subjects

The participants had to fast for at least 3 h before undergoing the fMRI scan, which was performed between 11:00 and 12:30 a.m. (before lunch). In the MRI scanner, the participants were positioned head-first supine. A mirror was fixed on the head coil above the participants at eye level to allow the visualization of an LCD screen at the rear of the scanner on which the visual task was presented. The participants were able to make choices with a response grip placed in their right hand. Before the task, the participants received the corresponding task instructions orally.

#### Magnetic Resonance Imaging Acquisition

Acquisitions were performed either on a 3T Siemens MRI (Magnetom Siemens Verio syngo MR B17, *N* = 28) using a 32-channel head coil or a 64-channel head coil (Magnetom Siemens Prisma syngo MR E11, *N* = 22). To avoid any difference specific to the magnet, we added a magnet covariable in all statistical analyses. The Nordic Neurolab solution (Bergen, Norway) and EPrime 2.0.8 Professional (PST, Sharpsburg, MD, United States) were used to display the visual task *via* a rear-facing mirror placed on the head coil and an LCD screen at the back of the MRI bore. An anatomical sequence (T1 3D MP-RAGE, FOV = 256 mm × 256 mm, 176 slabs, TR = 1,900 ms, TE = 2.26 ms, TI = 900 ms, flip angle = 9°, voxels size = 1 mm × 1 mm × 1 mm) and an axial T2 FLAIR sequence were acquired in order to detect a potential brain abnormality, in which case the subject was excluded from the analysis and referred to a specialist physician. Functional MRI with a sensitive T2-weighted gradient echoplanar imaging sequence with 36 slices of 3 mm was acquired during the 12-min cognitive task (350 volumes). The acquisition parameters were FOV = 192 mm × 192 mm, EPI factor 64, voxels size = 3 mm × 3 mm × 3 mm, TR = 2,000 ms, TE = 30 ms, and flip angle = 90°. Resting-state (rsfMRI) with a sensitive T2-weighted gradient echoplanar imaging sequence with 20 slices of 3 mm was acquired in 12 min (514 volumes). The acquisition parameters were FOV = 192 mm × 192 mm, EPI factor 64, voxel size = 2.3 mm × 2.3 mm × 4 mm, TR = 1,400 ms, TE = 30 ms, and flip angle = 70°.

#### Functional Magnetic Resonance Imaging Analysis

Image analysis was performed using Statistical Parametric Toolbox 12 (Wellcome Department of Imaging Neuroscience, London, United Kingdom) in MATLAB (Mathworks, Inc., Sherborn, MA, United States). Functional images were realigned to the mean to correct for motion during the acquisition and coregistered on 3D T1 anatomical images. The anatomical and functional images were then normalized to the Montréal Neurological Institute (MNI) space. Spatial smoothing of functional images was performed with a 6-mm full width at half maximum kernel (FWHM). Vector onsets were compiled for the SL, DL, HE, and LE conditions using MATLAB. A general linear model analysis was performed on brain masked data, using the canonical hemodynamic response function (HRF) without time and dispersion derivatives. The analyzed model was built with SL, DL, LE, and HE brain responses and the following covariables: PCA1 and PCA2 values and their interaction, magnet types, and, as recommended in Smeets et al. (29), orthogonalized values of hunger, thirst, wellbeing, and menstrual day. Statistical analysis was performed on all subjects as well as for PCA1 and PCA2 values and interactions with the following contrasts: (i) any choice, as defined from any brain responses in the SL and DL conditions, (ii) SL condition, (iii) DL condition, or (iv) SL vs. DL conditions. For the all-subject analysis, statistical significance was assessed for non-corrected *p*-value at a cluster peak level of *p* < 0.001 and family-wise error correction at the cluster level of pFWE < 0.05. For the analysis with the PCA1 and PCA2 values, a non-corrected value at a cluster peak level of *p* < 0.001 was considered.

#### Resting-State Functional Magnetic Resonance Imaging Analysis

Functional connectivity (FC) was performed using CONN Toolbox version 20.b on MATLAB 2017b environment (The MathWorks, Inc., Natick, MA, United States). After all preprocessing steps including normalization to MNI atlas space, spatial smoothing with an 8-mm FWHM, motion artifact detection, denoising, and first-level analysis, a seed-to-voxel second-level analysis were performed with the same covariates as for the task fMRI analysis (see above). The right nucleus accumbens was chosen as seed, given its detected activation during food choice, depending on the PCA1 value. The right insula was chosen as seed, given its detected activation during food choice, depending on the PCA2 value. Statistical significance was set upon Gaussian Random Field Theory parametric statistics, cluster threshold pFDR < 0.05 on cluster size, and a voxel threshold with a *p*-value < 0.001, uncorrected. rsfMRI acquisition was not performed for one individual due to missing acquisition.

### Statistics

Except for the brain imaging analysis described above, all statistical analyses were performed with the general linear model, an univariate analysis, or repeated measurement (SPSS software), with a threshold of *p* < 0.05 for statistical significance.

## Results

### Eating Habits

Based on the FCFQ scoring of the entire population (*n* = 178), the PCA first dimension (PCA1, explaining 22.39% of the response variability) was associated with an increased score of all palatable food categories, whereas the PCA second dimension (PCA2, explaining 13.92% of the response variability) was more oriented toward “sweet” and “protein” food items and could not explain the score of the sweet breakfast, ice cream, appetizer, and fat food categories (Figure 2A). The PCA1 values were used to select 50 individuals and to separate WTc from PTc. WTc had higher food frequency consumption of nearly all palatable food categories, ensuring a satisfactory selection process for the entire selected population (Table 1). Among the 50 selected individuals, the related PCA1 and PCA2 showed an association with the food-frequency of nearly all palatable food categories, such as that seen in the entire population. As seen in the selected (WTc and PTc) vs. non-selected participants in Figure 2B, and given the non-clear-cut separation between WTc and PTc along the PCA1, we chose to use the values of PCA1, PCA2, and their interaction for the brain analysis.

**TABLE 1:** Hedonic food frequency consumption for the entire population (*N* = 178) and the subpopulation (*N* = 50) selected for the brain imaging study.

	*N*	ColdMeat_Eggs	Meat	ProcessFood	Dessert	SweetBreakfast	Sweet	IceCream	Appetizer	Cheese	Fat	PCA1	PCA2
ALL	**178**	2.15 ± 1.78	2.98 ± 2.08	1.33 ± 0.92	9.94 ± 9.39	9.40 ± 7.76	3.53 ± 3.04	0.69 ± 0.85	2.20 ± 3.11	3.81 ± 3.33	4.72 ± 3.74	na	na
ALL-178													
PCA1, p-value		**<0.001**	**<0.001**	**<0.001**	**<0.001**	**<0.001**	**<0.001**	**<0.001**	**<0.001**	**<0.001**	**<0.001**		
PCA2, p-value		**<0.001**	**0.010**	0.338	**<0.001**	**<0.001**	**<0.001**	0.470	*0.074*	**<0.001**	0.349		
**Volunteers selection criteria, *n* = 50**													
PTc	**25**	1.75 ± 1.10	2.57 ± 1.85	1.11 ± 0.46	8.29 ± 6.91	7.69 ± 7.01	2.72 ± 2.55	0.44 ± 0.44	1.24 ± 1.09	3.04 ± 2.79	2.43 ± 2.07	−0.49 ± 0.66	0.29 ± 1.03
WTc	**25**	2.98 ± 1.89	3.85 ± 1.91	1.97 ± 1.28	12.39 ± 11.05	11.81 ± 8.28	4.95 ± 3.77	1.23 ± 1.22	5.08 ± 6.50	6.29 ± 4.16	8.57 ± 4.55	2.33 ± 1.47	0.38 ± 1.38
*p*-value	**na**	**0.006**	**0.02**	**0.003**	**0.122**	*0.064*	**0.018**	**0.004**	**0.005**	**0.002**	**<0.001**	**<0.001**	0.811
**Volunteers characterization, *n* = 50**													
PCA1, *p*-value		**0.040**	**0.001**	**<0.001**	**0.016**	0.201	**<0.001**	**<0.001**	**<0.001**	**0.001**	**<0.001**		
PCA2, *p*-value		**<0.001**	0.256	0.422	**<0.001**	**0.004**	**<0.001**	0.746	0.746	**0.007**	0.940		

*PCA, principal component analysis. PCA1 and PCA2 are dependent variables corresponding to the individual projection values on the first two axes of the PCA performed on the food frequency questionnaire data. PTc, prudent-type consumers (N = 25) with the highest values for PCA1 among the 50 selected volunteers. WTc, Western-type consumers (N = 25) with the lowest values for PCA1 among the 50 selected volunteers. na, not applicable. Values in bold are statistically significant for p < 0.05, values in italic are tendencies for 0.05 < p < 0.10.*

### Body Composition, Resting Metabolic Rate, and Behavioral Characterization

The description and details about the selected population are provided in Table 2. With the selection criteria based on the PCA1, the WTc and PTc populations were similar, except for BMI, with a significantly higher BMI in the PTc group, and the emotional component of eating from TFEQ, with a significantly higher emotional eating component in the WTc group. Interestingly, the PCA1 and the PCA2 axis explained two distinct components of eating measured with the TFEQ, whereas all other measurements for body composition, resting metabolic rate, and behavioral characterization were similar. PCA1 yielded information about the difference in emotional eating, whereas PCA2 yielded information about the difference in uncontrolled eating in the studied individuals.

**TABLE 2:** Characterization of the population selected for the brain imaging study (*N* = 50).

	Age (years)	Weight (kg)	Height (cm)	BMI (kg/cm^2^)	DER (kcal/day)	Fat (%)	Ricci-Gagnon	SCOFF	TFEQ: CR	TFEQ: EE	TFEQ: UE	EOQ	AUDIT	CRAFT
All selected, *n* = 50	20.3 ± 1.9	59.0 ± 5.9	167.0 ± 5.1	21.2 ± 1.9	1,382.1 ± 164.3	23.5 ± 5.9	19.2 ± 6.6	0.04 ± 0.20	8.7 ± 5.0	5.1 ± 2.7	13.4 ± 7.5	2.1 ± 2.5	3.1 ± 2.1	0.20 ± 0.45
PTc, *n* = 25	20.4 ± 2.0	60.0 ± 5.0	166.0 ± 5.7	21.8 ± 2.0	1360.2 ± 180.3	24.2 ± 5.1	19.5 ± 6.3	0.08 ± 0.28	8.6 ± 5.3	4.2 ± 2.3	12.4 ± 8.0	1.84 ± 2.9	2.8 ± 1.8	0.24 ± 0.52
WTc, *n* = 25	20.1 ± 1.8	58.0 ± 6.5	168.0 ± 4.3	20.5 ± 1.7	1403.2 ± 147.2	22.9 ± 6.7	19.0 ± 6.9	0.00 ± 0.00	8.9 ± 4.7	6.0 ± 2.9	14.4 ± 6.9	2.3 ± 2.0	3.4 ± 2.3	0.16 ± 0.37
*p*-value	0.272	0.257	0.176	**0.017**	0.352	0.430	0.763	0.155	0.845	**0.018**	0.357	0.497	0.346	0.537

**PCA axis effect**	**Age**	**Weight**	**Height**	**BMI**	**DER**	**Fat**	**Ricci-Gagnon**	**SCOFF**	**TFEQ: CR**	**TFEQ: EE**	**TFEQ: UE**	**EOQ**	**AUDIT**	**CRAFT**

PCA1, *p*-value	0.346	0.852	0.182	0.234	0.508	0.792	0.668	0.520	0.986	**0.020**	0.384	0.425	*0.061*	0.316
PCA2, *p*-value	0.595	0.901	0.400	0.662	0.813	0.949	0.977	0.637	0.169	0.345	**0.007**	0.520	0.603	0.332

*PCA, principal component analysis. PCA1 and PCA2 are dependent variables corresponding to the individual projection values on the first two axes of the PCA performed on the food frequency questionnaire data. PTc, prudent-type consumers (N = 25) with the highest values for PCA1 among the 50 selected volunteers. WTc, Western-type consumers (N = 25) with the lowest values for PCA1 among the 50 selected volunteers. BMI, body mass index; BMR, basal metabolic rate; fat, body fat composition assessed with a body impedance meter; Ricci-Gagnon, questionnaire assessing physical activity level; SCOFF, TFEQ: Three-factor Eating Questionnaire; CR, cognitive restriction; EE, emotional eating; UE, uncontrolled eating; EOQ, Emotional Overeating Questionnaire; AUDIT, questionnaire assessing drinking patterns; CRAFT, questionnaire assessing substance-related risks and problems. Values in bold are statistically significant for p < 0.05.*

### Brain Responses During Functional Magnetic Resonance Imaging Wanting Task

We could not detect any difference in the reaction time and in the response number that could be explained by PCA1 or PCA2. Only thirst and hunger were associated with a limited effect in the reaction time and in the HE vs. LE response number (Supplementary Table 1). Note that one outlier individual was removed from the analysis since the number of LE vs. HE responses, and the reaction times were out of range of the group average ± 2 SD. This individual was further removed for the fMRI wanting task (*n* = 49) and rsfMRI FC (*n* = 48) analysis.

**FIGURE 3:**
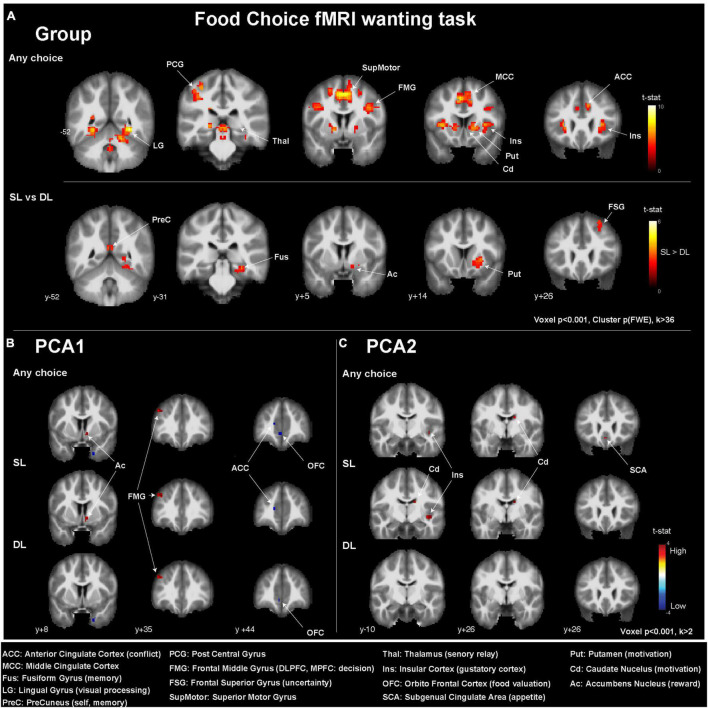
Brain activations detected in the fMRI two-choice task viewed in coronal slices **(A)** for all participants (*n* = 50), **(B)** depending on the PCA1 values, and **(C)** depending on PCA2 values. MNI *y*-coordinates are indicated for each coronal slice. ACC, anterior cingulate cortex; MCC, middle cingulate cortex; Fus, fusiform gyrus; LG, lingual gyrus; PreC, precuneus; PCG, posterior central gyrus; FMG, frontal middle gyrus; FSG, frontal superior gyrus; SupMotor, superior motor gyrus; Thal, thalamus; Ins, insular cortex; OFC, orbitofrontal cortex; SCA, subgenual cingulate area; Put, putamen; Cd, caudate nucleus; Ac, accumbens nucleus.

In all individuals and for any choice condition (Figure 3 and Table 3), i.e., during SL and DL conditions, we could detect some activated clusters in the brain regions associated with food choices, the gustatory cortex (insula: Ins), the motivation centers (caudate: Cd, putamen: Put), and also in conflict monitoring brain region (anterior cingulate cortex, ACC) and the thalamus (sensory relay, regulation of consciousness, and alertness) as seen in the activation map (Figure 3A). When comparing SL condition vs. DL condition, we could detect higher brain responses in the reward system (right nucleus accumbens: Ac) and the motivation center (right Put).

**TABLE 3:** Functional magnetic resonance imaging wanting task for the second-level analysis in all individuals.

fMRI wanting task, *n* = 49^a^		Anatomical area		*p*-Value (cluster)			MNI peak coordinates			*p*-Value (peak)	
					
	*K*	Structure	Hemisphere	FWE	FDR	Uncorr	*x*	*y*	*z*	*T*	Uncorr
ALL: SL vs. DL conditions	108	Fusiform, *k* = 42	R	<0.001	0.001	<0.001	30	−31	−16	4.97	<0.001
	89	Putamen, *k* = 43	R	0.001	0.001	<0.001	24	11	−4	4.67	<0.001
		Accumbens, *k* = 2	R				15	8	−10	3.85	<0.001
	77	Precuneus, *k* = 28	L	0.002	0.002	<0.001	0	−58	17	4.65	<0.001
	47	Frontal sup area, *k* = 29	R	0.021	0.021	0.001	27	26	56	4.25	<0.001

**rsfMRI - functional connectivity, *n* = 48^b^**		**Anatomical area**		***p*-value (cluster size)**			**MNI peak coordinates**			***p*-Value (peak)**	
					
	***K***	**Structure**	**Hemisphere**	**FWE**	**FDR**	**Uncorr**	***x***	***y***	***z***	**Uncorr**	

PCA1, right accumbens as seed	252	Orbitofrontal cortex, *k* = 120	L	0.003	0.002	<0.001	−44	52	−12	<0.001	
		White matter, *k* = 6	L								
PCA2, right insula as seed	226	Anterior cingulate cortex, *k* = 22	L/R	0.008	0.007	<0.001	−6	16	20	<0.001	
		Corpus Callosum, *k* = 190	L/R								

*^a^Height threshold T = 5.52, pFWE < 0.05, and pUncorr < 0.001 at peak level, df = 1.42, extent threshold: k = 30 voxels, voxel size: 3.0 mm × 3.0 mm × 3.0 mm.*

*^b^Height threshold T = 3.55, pFDR < 0.05 at cluster level, pUncorr < 0.001 at peak level, df = 1.40, extent threshold: k > 125 voxels. L, left; R, right; K, cluster size (number of voxels); SL, similar liking; DL, different liking; FWE, family-wise error correction; FDR, false discovery rate correction; Uncorr, uncorrected p-value.*

When investigating the effect of the PCA axis value on the brain response during the wanting task, only uncorrected statistics (*p* < 0.001) were considered. The PCA1 was associated with higher brain responses in (i) the Ac for all choices and SL conditions, (ii) the frontal middle gyrus (FMG), and more precisely the dorsolateral prefrontal cortex (DL-PFC), for all conditions and, with lower brain response in (i) the ACC for all choices and SL condition and (ii) the orbitofrontal cortex (OFC) for all choices and the DL condition. The PCA2 was associated with higher brain responses in (i) the Cd and Ins for all choices and SL conditions, as well as (ii) the subgenual cingulate area (SCA) for all choices only.

### Resting-State Functional Magnetic Resonance Imaging-Based Functional Connectivity

**FIGURE 4:**
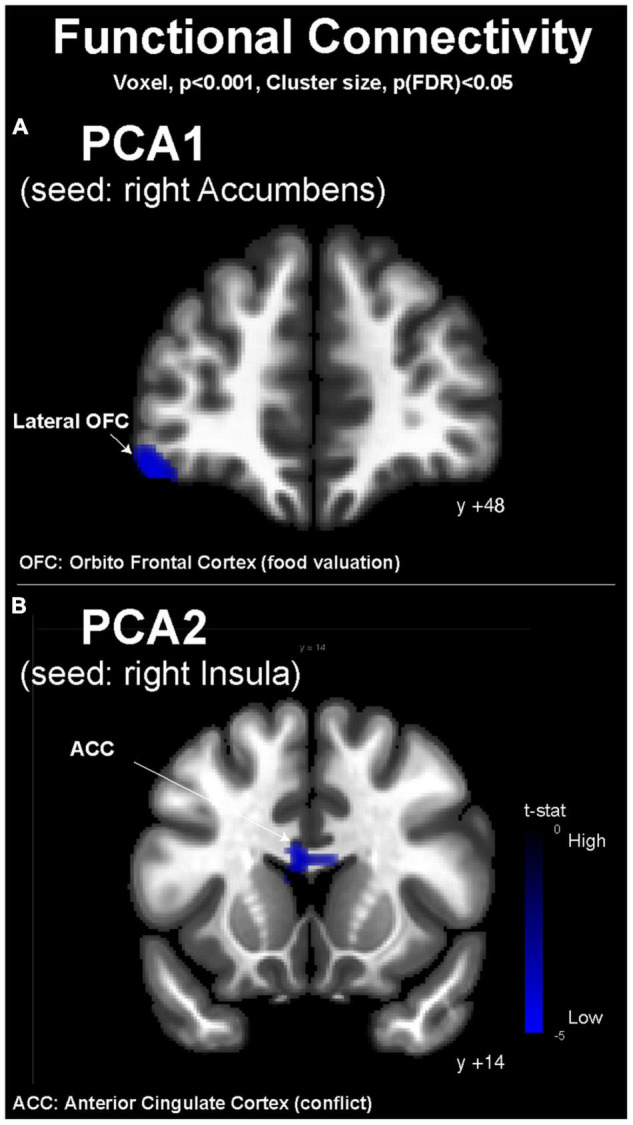
Resting-state functional magnetic resonance imaging functional connectivity maps (conn toolbox). **(A)** Depending on the PCA1 values with the right accumbens as seed and **(B)** depending on PCA2 values with the right insula as seed. MNI coordinates are indicated for each coronal slice. OFC, orbitofrontal cortex; ACC, anterior cingulate cortex.

Building on the brain response during the fMRI wanting task, we used as seeds for FC analysis (i) the right Ac to investigate the effect of PCA1, and (ii) the right Ins to investigate the effect of PCA2. The PCA1 was associated with an inverse correlation with the FC between the right Ac and the left lateral OFC (Figure 4A). The PCA2 was associated with an inverse correlation with the FC between the right Ins and the ACC (Figure 4B).

## Discussion

In this study, we aimed to select, in a well-characterized healthy normal-weight population, two distinct subpopulations of young women based on their food frequency profiles, either WTc or PTc. We first described their differences in terms of body composition and eating behavior before discussing their brain responses during an fMRI food wanting task as well as during rest for FC analysis between several brain regions of interest, for which anomalies have been previously described in the context of obesity and EDs (16, 19).

### Different Hedonic and Consumption Profiles Are Associated With Specific Eating Behavior Components and Risks

Despite the strict selection criteria used to homogenize our population while excluding EDs, food addiction (30), and other confounding factors, we managed to identify two subgroups based on their hedonic profiles and consumption frequency of Western-type food items, i.e., WTc vs. PTc. The sample of 50 young participants in our brain imaging study was fairly representative of the entire studied population of 178 young women in terms of hedonic food consumption. The two subgroups only differed on the basis of their BMI, which was higher in PTc than in WTc, and emotional eating, which was higher in WTc than in PTc. After selection, we decided to further investigate this population sample in line with the two first PCA axis values. The PCA1 axis was associated with a Western diet (WD) eating profile, i.e., higher consumption of transformed, sweet, and fatty food items, whereas the PCA2 axis was associated with WD excluding fatty food item consumption. Both axes were not associated with differences in the participants’ age, weight, height, BMI, BMR, percentage of body fat, physical exercise, EDs, emotional overeating, alcohol consumption, or substance abuse. However, the PCA1 axis was associated with the emotional component of eating, whereas the PCA2 axis was associated with the uncontrolled component of eating, as observed with the TFEQ results. This suggests that, within a homogenous population of normal-weight and healthy young women, specific hedonic profiles based on food consumption declaration can already be connected with specific trends in eating behavior and, especially, risky components in terms of eating habits. The question of whether the individual trajectories are already well-established or might change with time and how flexible they can be remains.

### Brain Functions Are Influenced by Hedonic and Food Consumption Profiles

Brain imaging techniques, especially fMRI and rsfMRI, represent potent tools to investigate eating behavior and its neural correlates (16). In this study, we used (17) an fMRI wanting task (28) in order to investigate the brain responses during more or less conflicting food choices. One hypothesis related to this task is that a choice between two food items with different energy densities but SL would induce less cognitive/decision conflict than a choice between two food items with DL, the high-liking food item always being the one with higher energy density in our task design. Indeed, in this situation, people who tend to make food decisions on the basis of health preoccupations would encounter a cognitive conflict between the food item’s healthiness and palatability. However, another hypothesis can present the problem on a different level. People who tend to make food decisions on the basis of their hedonic preferences would rather have more difficulties choosing between two dishes that are similarly preferred. Then, it is likely that the SL situation can represent either a more or less conflicting choice than the DL depending on the individual mindset and priorities. The significantly higher reaction time for the SL situation than the DL situation (1,524 vs. 1,339 ms) might indicate that SL represented a more conflicting choice, i.e., subjects needed more time to take a decision when they were confronted with two food items similarly preferred. This is where brain imaging can help going further in the interpretation of subtle cognitive decisions.

In the overall selected population (*N* = 50), we detected significant brain responses to the task in gustatory centers, i.e., brain regions involved in food motivation and reward, decision-making, and conflict resolution. The PCA axes delivered little information about specific brain responses related to hedonic food consumption. This could be explained by the limited population sample. We observed subtle changes in brain activity related to PCA1 and PCA2. PCA1 was positively associated with the FMG activity, specifically in the DL-PFC, which is related to decision-making and cognitive control. Only in the SL condition, PCA1 was positively associated with the ventral striatum (Ac) activity and negatively associated with the ACC activity, involved in conflict resolution. Only in the DL condition, PCA1 was negatively associated with the OFC activity, involved in food hedonic valuation. Consequently, differences in WD food consumption in a normal-weight and healthy young population can already shape the brain responses toward food choices and the type of cognitive conflict encountered during such decisions. The brain networks that are differently modulated during food choices and decisions might be of interest as potential early indicators of risky habits or EDs (31).

Because the nucleus accumbens (Ac) is considered a hedonic hotspot (32), we investigated its FC during the resting state in relationship with the hedonic and consumption profiles of our volunteers, represented by the PCA1 values. PCA1 presented an inverse association with FC between Ac and the lateral OFC, suggesting that people who choose and consume WD food items more frequently present less FC between the OFC, involved in the hedonic valuation and stimulus–reward association, and the Ac, where food reward is perceived (31). This result is particularly interesting because it suggests that the neurocognitive process of decision-making is different and perhaps less efficient in high consumers than in low consumers of Western-type food items. Interestingly, in the fMRI wanting task, the PCA2 showed different associations with brain activity compared with PCA1. In the SL condition, PCA2 was associated with a modulated activity in the gustatory cortex (right Ins) and in the Cd involved in food motivation. We consequently put further investigation on the right Ins and its resting-state FC in relationship with the PCA2 values. PCA2 presented an inverse association with FC between the right Ins and the ACC, suggesting that people who choose and consume WD food items more frequently, except for fatty foods, present less connectivity between the motivation center and the ACC, which is involved in goal-oriented attention and conflict resolution (33, 34). This distinction between PCA1 and PCA2 might illustrate two different subsets of WD profiles, one characterized by the high consumption of all food items classically associated with WD (PCA1) and the other characterized by high consumption of meat, processed foods, and sweet foods, but not fatty foods. Some authors describe addiction to fat-rich diets as a prominent subset of food addiction (35), but our results on PCA1 show that people who consume fatty foods also consume high quantities of meat, processed foods, and sweet foods, while some others (high PCA2 values) present consumption of fatty foods that are not correlated with the consumption of other typical WD items. A continuum might exist in WD profiles, with intermediate phenotypes or subsets of WD eaters for whom different nutritional counseling and guidance might be proposed. More important is the fact that these hypothetical subsets of WD eaters present different characteristics in terms of neurocognitive control of food intake and valuation, with possibly different weaknesses in relation to conflict monitoring, decision-making, or goal-oriented motivation. These observations and knowledge are critical to designing better interventions for prevention and treatment, including behavioral and cognitive therapy with or without brain imaging guidance.

### Toward the Definition of Early Risk Factors and Individual Health Trajectories

In this study, we aimed at identifying some neural mechanisms that could explain, on the basis of eating behavior and regular consumption of Western-type food items, the early shift in hedonic and cognitive processes that might instill and even worsen risky habits in normal-weight healthy young adults and consequently favor the onset of EDs and weight gain. On the basis of PCA1 and PCA2, we showed that different subsets of WD eaters possibly exist and are associated with different components of eating behavior and neurocognitive responses. PCA2 was indeed associated with the consumption of WD food items without the fat component as well as with uncontrolled eating. The fMRI wanting task showed that these characteristics influenced the responses of the gustatory cortex and the motivation center during food choices. These characteristics also influenced the resting-state FC between the gustatory cortex and ACC, possibly revealing an impact of individual habits and preferences on food-related conflict resolution. PCA1, which explains the consumption of almost all WD food items, was associated with the emotional component of eating modulation, possibly revealing an anomaly in emotion regulation toward food in high consumers of WD food items. This is in line with a large bundle of evidence illustrating the tight connections between emotion regulation and eating behavior (31) or between diet and related disorders such as depression (36). The fMRI wanting task results suggest that the PCA1 characteristics are associated with a modulation of the reward system and decision-making processes. The FC between these networks is also modulated. As a consequence, there is probably a tight reciprocal connection between individual eating habits and specific top-down cognitive control processes that might determine the individual trajectories in terms of health and diseases. Interestingly, obesity is characterized by reduced FC within the brain circuitry involved in cognitive control and the reward system, notably between cortical and striatal brain regions (19). Our study lacks longitudinal measurements in order to validate this hypothesis and the causal relationship between eating habits and specific neurocognitive processes. Given that we tried to investigate subtle changes in a rather homogenous yet limited sample of volunteers, further studies should consider increasing the number and diversity of participants. Specific interventions, either on eating habits (e.g., through nutritional counseling) or on neurocognitive processes (e.g., through behavioral and cognitive therapies or neuromodulation strategies), might also help decipher the respective and possibly bilateral influences of one and the other.

## Conclusion

In conclusion, this study brings to the front interesting relationships between the consumption of WD food items, specific eating behavior components, and brain functions, such as those involved in the cortico-striatal circuitry dedicated to cognitive control, in a homogenous population of normal-weight healthy young adults. Early identification of risky eating habits and/or anomalies in the underlying neurocognitive mechanisms might be used to extrapolate individual trajectories in terms of health problems, such as weight gain or the onset of EDs. Ultimately, these explorations are also valuable to identify the best individual strategy to implement in order to correct eating habits and/or cognitive control.

## Data Availability Statement

The datasets for this study can be found in the INRAE open-access repository: 10.57745/ULUDGI.

## Ethics Statement

The studies involving human participants were reviewed and approved by the Comité de Protection des Personnes “Ile de France II,” project N° 2017-03-03; N° ID-RCB/EUDRACT 2017-A00133-50; National Clinical Trial number: NCT03076489. The patients/participants provided their written informed consent to participate in this study.

## Author Contributions

DV-L secured funding for this project. NC, DV-L, RM, AC, and PM designed the research. PM built the food picture database. YG built the screening questionnaire and recruited participants. NC, YS, PM, and EB contributed to the fMRI task setup, fMRI acquisition, and fMRI analysis. AC created the numeric form of the Food Consumption Frequency Questionnaire, Liking, and Caloric numeric scale questionnaires, performed on TypeForm^®^. RT provided the devices and methodology for body composition and resting energy expenditure measurements. YG, YS, PM, NC, and DV-L performed the research. NC analyzed the data and produced the brain activations illustrations. NC and DV-L wrote the manuscript. All co-authors read and revised the manuscript. All authors contributed to the article and approved the submitted version.

## Conflict of Interest

The authors declare that the research was conducted in the absence of any commercial or financial relationships that could be construed as a potential conflict of interest.

## Publisher’s Note

All claims expressed in this article are solely those of the authors and do not necessarily represent those of their affiliated organizations, or those of the publisher, the editors and the reviewers. Any product that may be evaluated in this article, or claim that may be made by its manufacturer, is not guaranteed or endorsed by the publisher.

## Abbreviations

ACC: anterior cingulate cortex
AUDIT: Alcohol Use Disorders Identification Test
BMI: body mass index
BOLD: blood oxygen level-dependent
CRAFFT: Car Relax, Alone Forget, Friends Trouble
DL: different liking
DL-PFC: dorsolateral prefrontal cortex
EDs: eating disorders
EOQ: Emotional Overeating Questionnaire
FC: functional connectivity
FCFQ: food consumption frequency questionnaire
FG: fusiform gyrus
fMRI: functional magnetic resonance imaging
HE: high energy
OFC: orbitofrontal cortex
LE: low energy
PCA: principal component analysis
PCA1: PCA-projected values on the first axis
PCA2: PCA-projected values on the second axis
PTc: prudent-type consumers
rsfMRI: resting-state functional magnetic resonance imaging
SCOFF: Sick Control, One stone Fat Food
SL: similar liking
TFEQ-R18: Three-Factor Eating Questionnaire-Revised 18-item
WD: Western diet
WTc: Western-type consumers.
