# Supplementary material for: Brain Responses to Food Choices and Decisions Depend on Individual Hedonic Profiles and Eating Habits in Healthy Young Women
Source: Front Nutr. 2022 Jun 24;9:920170. doi: 10.3389/fnut.2022.920170 (PMC9263555; doi:10.3389/fnut.2022.920170)
Supplement: Supplementary file 1 [file Table_1.DOCX]

| **Supplementary Table 1:** fMRI wanting task, reaction time and response number | | | | | | |  |  |  |  |  |  |  |  |  |  |  |  |  |
| --- | --- | --- | --- | --- | --- | --- | --- | --- | --- | --- | --- | --- | --- | --- | --- | --- | --- | --- | --- |
|  |  | **Reaction time (ms)** | |  |  |  |  |  |  |  | **Response number** | |  |  |  |  | **choice ratio** |  |  |
|  |  | **SL** | **DL** | **HE Choice** | **LE Choice** | **SL:HE** | **SL:LE** | **DL:HE** | **DL:LE** |  | **HE Choice** | **LE Choice** | **SL:HE** | **SL:LE** | **DL:HE** | **DL:LE** | **HE/LE** | **SL:HE/LE** | **DL: HE/LE** |
| **ALL, n=49** | **m** | 1524 | 1339 | 1419 | 1460 | 1511 | 1545 | 1330 | 1378 |  | 51.14 | 38.84 | 25.02 | 19.96 | 26.12 | 18.88 | 1.49 | 1.40 | 1.70 |
|  | **sd** | 250 | 250 | 253 | 251 | 251 | 273 | 271 | 257 |  | 9.19 | 9.16 | 4.61 | 4.58 | 5.63 | 5.63 | 0.90 | 0.75 | 1.25 |
| **WTc vs PTc** |  | ns | | | | | | | |  | ns | | | | | | | | |
| **Hunger** |  | ns | | | | | | | |  | 0.045 | 0.043 | ns | ns | *0.051* | *0.051* | *0.054* | ns | *0.051* |
| **Menstrual Day, Thirst, Well-Being, PCA1, PACA2** |  | ns | | | | | | | |  | ns | | | | | | | | |
|  |  | **Reaction time (ms)** | |  |  |  |  |  |  |  | **Response number** | |  |  |  |  |  |  |  |
|  |  | **SL vs DL** | | **HE vs LE** | | **SL:HE vs SL:LE** | | **DL:HE vs DL:LE** | |  | **HE vs LE** | | **SL:HE vs SL:LE** | | **DL:HE vs DL:LE** | |  | **SL:HEL/LE vs DL:HE/LE** | |
| **ALL, n =49** |  | <0.001 | | 0.091 | | ns | | ns | |  | <0.001 | | 0.001 | | 0.001 | |  | 0.072 | |
| **WTc vs PTc** |  | ns | | | | | | | |  | ns | | | | | | | | |
| **Hunger** |  | ns | | 0.038 | | ns | | 0.033 | |  | 0.044 | | ns | | 0.051 | |  | ns | |
| **Thirst** |  | ns | | 0.016 | | 0.052 | | 0.093 | |  | ns | | ns | | ns | |  | 0.021 | |
| **Menstrual Day, Well-Being, PCA1, PCA2** |  | ns | | | | | | | |  | ns | | | | | | | | |
